# Exercise in Glucose-6-Phosphate Dehydrogenase Deficiency: Harmful or Harmless? A Narrative Review

**DOI:** 10.1155/2019/8060193

**Published:** 2019-04-04

**Authors:** Kalliopi Georgakouli, Ioannis G. Fatouros, Dimitrios Draganidis, Konstantinos Papanikolaou, Panagiotis Tsimeas, Chariklia K. Deli, Athanasios Z. Jamurtas

**Affiliations:** ^1^Department of Physical Education and Sport Science, University of Thessaly, Karies, Trikala 42100, Greece; ^2^Institute of Human Performance and Rehabilitation, Centre for Research and Technology Thessaly, Karies, Trikala 42100, Greece

## Abstract

**Objectives:**

Glucose-6-phosphate dehydrogenase (G6PD) deficiency, theoretically, renders red blood cells (RBC) susceptible to oxidative stress. G6PD deficiency has also been found in other types of cells than RBC, such as leukocytes and myocytes, where an inefficient protection against oxidative stress may occur too. Glutathione (GSH), a significant antioxidant molecule, levels are lower in G6PD individuals, and theoretically, the probability of oxidative stress and haemolysis due to exercise in individuals with G6PD deficiency is increased, whereas dietary supplementation with antioxidants may have beneficial effects on various aspects of this enzymopathy.

**Methods:**

A search of the available literature was conducted using the keywords glucose-6-phosphate dehydrogenase (G6PD), deficiency, disease, exercise, muscle, antioxidant, vitamin, supplement, and supplementation. The search was limited to publications in English, conducted on humans, and published until August 2018. After screening, only relevant articles were included.

**Results:**

There is little evidence indicating that G6PD deficiency can cause perturbations in redox status, haemolysis, and clinical symptoms such as fatigability and myoglobinuria, especially after intense exercise, compared to individuals with normal enzyme levels.

**Conclusions:**

Exercise could be used by G6PD-deficient individuals as a tool to improve their quality of life. However, there is a lack of training studies, and assessment of the effects of regular and systematic exercise in G6PD-deficient individuals is warranted. Finally, since GSH levels are lower in G6PD deficiency, it would be interesting to examine the effects of antioxidant or cysteine donor supplements on redox status after exercise in these individuals.

## 1. Introduction

Glucose-6-phosphate dehydrogenase (G6PD) is an enzyme that helps cells to counterbalance oxidative stress, which is an imbalance between antioxidant defence (enzymatic and nonenzymatic) and the production of reactive oxygen and nitrogen species (RONS) in favour of RONS. Among other functions, G6PD is involved in the regeneration of reduced glutathione (GSH) from its oxidized form (GSSG), a reaction catalysed by glutathione reductase [1]. GSH is a major endogenous antioxidant that protects cells against oxidative damage in several ways. Therefore, G6PD-deficient activity, and consequently lower GSH levels, theoretically, renders cells susceptible to oxidative stress.

G6PD deficiency is a genetic disorder and the most common enzymopathy, affecting more than 400 million people worldwide [2]. More than 300 G6PD variants have been identified and are responsible for different levels of the enzyme activity. The World Health Organization (WHO) classifies these variants into five classes, according to the level of residual enzyme activity and the associated clinical manifestations [2]. Although in most cases G6PD deficiency causes no symptoms, it may lead to diseases such as neonatal jaundice (hyperbilirubinemia) and acute or chronic haemolysis [3] as red blood cells (RBC) are susceptible to oxidative stress and consequently destruction [4]. Frequency and severity of haemolysis vary depending on the G6PD variant and exposure to various oxidative agents [2, 3, 5–7]. Such agents include oxidative drugs, chemicals, foods (fava beans), and even some vitamins (e.g., vitamin K). Moreover, infection is thought to be another common cause of haemolytic anaemia in G6PD-deficient individuals [8, 9]. Redox imbalance has been suggested to contribute to viral replication and virulence [10–12], and haemolytic episodes have been reported after various viral and bacterial infections in G6PD-deficient individuals.

There is a growing concern that G6PD deficiency may be involved in the pathogenesis of various diseases mediated by oxidative stress. Although G6PD deficiency is well studied in RBC, there is a paucity of information about the activity of G6PD in other cells such as white blood cells and myocytes. Prevalence of diseases and associated complications such as neonatal jaundice, diabetic ketoacidosis, acute renal failure, cataract, and cancer have been claimed to be increased among G6PD-deficient individuals, suggesting that G6PD deficiency may be a risk factor for a wide range of pathological conditions.

Reports indicate that G6PD deficiency is present in both RBC and the muscle and that a positive relationship exists between the two tissues in G6PD activity [13]. Since the musculature is heavily utilized during exercise and the generation of reactive oxygen species can over exceed the theoretically reduced antioxidant capacity of the G6PD-deficient individual, exercise could have harmful effects on various health aspects in these individuals. Furthermore, since RBC are extremely active during intense exercise [14], G6PD-deficient RBC might not be able to withstand the exercise-induced oxidative stress, and acute haemolytic anaemia could develop. On the other hand, it is well known that exercise confers multiple protective effects on the human body. Thus, advising G6PD-deficient individuals to refrain from participating in exercise without substantial evidence of the harmful effects of exercise to them does not seem prudent. The purpose of this review was to examine whether exercise has any positive or negative effects on individuals with G6PD deficiency. We believe that the topic is very interesting since it relates with the most common enzymopathy and could generate interest for more research that could potentially lead to exercise guidelines being formulated for these individuals.

## 2. Materials and Methods

A search of the available literature was conducted in the following databases: PubMed, Scopus, and Google Scholar. The keywords used were glucose-6-phosphate dehydrogenase (G6PD), deficiency, disease, exercise, muscle, antioxidant, vitamin, supplement, and supplementation. The search was limited to publications in English, conducted on humans, and published until August 2018. Then, the authors screened the abstracts of the identified articles to remove those that were obviously irrelevant. Studies that included exercise as a stimulus to generate perturbations on health, redox status, and performance indices were included.

## 3. Results

The literature search revealed a few case report studies on G6PD deficiency and its association with muscle G6PD activity and exercise (Table 1). Two of these articles report cases of G6PD individuals with clinical symptomatology following exercise [15, 16] whereas the other one, which describes the case of a world class runner, did not report any adverse results.

Table 2 summarizes results from five experimental studies that examined the effects of exercise on various haematological and/or blood redox status indices [18–22]. None of these methodologically sound experimental studies report unwanted results following different modes and types of exercise.

## 4. Discussion

Although G6PD deficiency in RBC has gained a lot of attention and a number of studies have enlightened many aspects of this enzyme defect, research on the deficient activity of G6PD in muscle cells is scarce. Bresolin and colleagues [15] investigated G6PD activity in muscle biopsy specimens of four G6PD-deficient patients with the Mediterranean variant. All patients were found to have a very low G6PD activity in the muscle and other tissues. Also, G6PD activity in myoblasts, myotubes, and skin fibroblasts of three patients was tested and found to be residual. These findings reinforced the view that G6PD deficiency is present not only in RBC but also in several cell types, while muscle G6PD deficiency was reported for the first time. Concerning muscle G6PD deficiency, a relation with muscular symptomatology after exercise was indicated; however, only one patient developed myoglobinuria after intense exercise, while another patient reported moderate exercise intolerance and recurrent episodes of myalgia and muscle fatigability. Despite the heterogeneity and small number of subjects, a first concern on the involvement of muscle G6PD deficiency in clinical symptoms after exercise was raised. Adding to this concern is the well-documented significant perturbation on redox status following exercise and sports involvement. In theory, since G6PD-deficient individuals are susceptible to oxidative stress and exercise results in excess of RONS generation, G6PD-deficient individuals would be more predisposed to RONS cell destruction and therefore should avoid performing heavy physical exercise [23].

During strenuous physical exercise, there is accelerated production of RONS in exercising muscles, heart, and other tissues [23], which can cause damage to biomolecules (DNA, protein, and lipids) and fatigue. A major antioxidant molecule of great importance for RBC and other animal cells is GSH. As mentioned before, G6PD-deficient individuals have lower levels of GSH in RBC and other cells, and they may be predisposed to increased oxidative stress. As mentioned earlier, there are reports in the literature that come from case studies with no rigid research designs, which report that heavy exercise in G6PD-deficient individuals caused clinical signs of haemolysis, muscle degeneration, myalgia, and myoglobinuria, which may be attributed to increased oxidative stress [15, 16, 24–26]. However, more recent and better methodologically designed experimental studies indicate that different intensities of exercise do not cause oxidative stress or haemolysis in G6PD-deficient individuals to a greater extent than expected for their nondeficient counterparts [18–22].

### 4.1. Exercise-Induced Oxidative Stress in G6PD-Deficient Individuals

The responsible mechanisms for the oxidative stress observed after exercise, especially that of high intensity, have been extensively discussed elsewhere [27]. Regarding G6PD-deficient individuals, there is some concern about the possibility of negative effects of intense exercise on health due to their increased susceptibility to oxidative stress; however, research on this topic is scarce and does not support that concern. Nikolaidis and colleagues [18] examined oxidative stress after exercise until exhaustion in G6PD-deficient individuals. Nine G6PD-deficient males and nine controls matched for age and maximal oxygen consumption (VO_2_max) participated in two exhaustive treadmill exercise protocols of different duration. The first trial lasted for about 12 min where VO_2_max was determined, and the second trial lasted for about 50 min (45 min at 70-75% VO_2_max and then at 90% till exhaustion) with a 7-day washout period between the two trials. GSH, GSSG, haematocrit (Hct), and haemoglobin (Hb) were significantly lower in the G6PD-deficient group than the control group at the baseline. After the two exercise protocols, Hct and Hb levels did not change, GSH decreased, and thiobarbituric acid reactive substances (TBARS), protein carbonyls (PC), catalase, and total antioxidant capacity (TAC) increased in both groups. GSSG increased and the GSH/GSSG ratio decreased after the long-duration trial in both groups. The results of this study showed that exercise until exhaustion did not lead to higher oxidative stress and increased haemoglobin oxidation in G6PD-deficient males in comparison to their normal counterparts, despite the lower baseline GSH levels in G6PD-deficient males.

The effect of moderate intensity exercise on markers of oxidative stress and haemoglobin oxidation in G6PD-deficient males was studied by Jamurtas and colleagues [19]. Nine males with G6PD deficiency and nine age-matched control males run at approximately 75% of their maximum heart rate (MHR) for 45 min. GSH and Hct were significantly lower in the G6PD-deficient group compared to the control group at the baseline. No changes in GSH, GSSG, GSH/GSSG, or lipid peroxides were found for any group after exercise. Also, Heinz body formation (an index of haemolysis) was not detected before or after exercise in either group. The results from this study indicate that moderate intensity exercise at approximately 75% MHR may not cause oxidative stress or haemolytic anaemia in G6PD-deficient individuals.

In another study from the same laboratory, Theodorou and colleagues [20] investigated the effect of high-intensity muscle-damaging exercise on markers of muscle function, blood redox status, and haemolysis in G6PD-deficient males. Nine G6PD-deficient males and nine control males matched for age and maximal isometric torque performed an isokinetic eccentric contraction session of the knee extensors of both legs. GSH and GSSG were lower in the G6PD-deficient group compared to the control group at the baseline. Immediately and till 5 days after exercise, changes in indices of muscle function, redox status, and haemolysis in the G6PD-deficient group were similar to those in the control group. GSH and GSSG were always lower in the G6PD-deficient group. These results suggest that high-intensity muscle-damaging exercise may not cause different fluctuation pattern on muscle function, blood redox status, and haemolysis in G6PD-deficient males in comparison to their normal counterparts. Finally, an experimental study reported that 30 min of moderate intensity (70-75% of VO_2_max) did not perturbate redox status indices (TBARS, GSH, and GSSG) in a G6PD male individual [22]. However, it has to be mentioned that this individual was well-trained since his VO_2_max was more than 57 ml/kg/min.

Therefore, the limited literature of experimental studies indicates that exercise does not seem to perturbate the redox status of G6PD-deficient individuals to a greater extent than individuals with normal enzyme levels. Furthermore, even though earlier case studies indicated an increased probability for increased haemolysis, more recent experimental studies do not support such findings. Even though it seems that there is less chance for haemolysis with intense exercise, it is unknown whether the assumed compromised ability of the immune system of the exercising G6PD-deficient individual will lead to increased predisposition for upper respiratory tract infection under stressful conditions. Also, it has to be mentioned here that there is a complete lack of studies that have examined the exercise training effects on redox and health status in general on G6PD-deficient individuals.

Finally, other factors that should be taken into consideration are the coexistence of another red blood cell defect (e.g., sickle cell trait) [16], and the G6PD variant of the studied subjects as different variants cause different levels of enzyme activity in various types of cells. All these factors could potentially render RBC more susceptible to oxidative stress, resulting in severe haemolysis after intense exercise.

### 4.2. Supplementation and Exercise in G6PD-Deficient Individuals

Exposure of G6PD-deficient individuals to oxidative stress, such as consumption of fava beans, use of certain drugs, and maybe infections, can lead to haemolytic anaemia [2]. Drug-induced haemolysis is thought to be the most common adverse clinical consequence of G6PD deficiency. For that reason, G6PD-deficient individuals are advised to avoid certain drugs. Theoretically, the lower levels of GSH in G6PD-deficient individuals could make them more susceptible to increased oxidative damage due to lower inherent antioxidant activity.

Regarding the use of dietary supplements, only a few studies have examined their effects on various health aspects in this population. A systematic review by Lee and colleagues [28] concluded that the use of herbal or dietary supplements at therapeutic doses is not likely to induce haemolysis in G6PD-deficient individuals. From the 10 dietary or herbal substances that were assessed, only henna (Lawsonia inermis) was found to increase the possibility for haemolysis [28]. In addition to that, dietary supplements may help in the prevention or improvement of haemolytic anaemia, as well as other adverse effects of oxidative stress.

Cysteine and other sulphur-containing compounds may result in increased levels of GSH; therefore, it could be hypothesized that supplementation with such compounds would restore GSH redox status in G6PD-deficient individuals, making their cells less susceptible to oxidative stress. Four weeks of supplementation with *α*-lipoic acid, a sulphur-containing compound and potent antioxidant, has shown to improve some indices of redox status in G6PD-deficient individuals with no signs of haemolytic anaemia [29]. Moreover, vitamin E may protect the membranes of RBC from oxidation and consequent haemolysis. Dietary supplementation of 800 IU of vitamin E per day for 3-12 months resulted in improvement of indices of chronic haemolysis in G6PD-deficient individuals [30, 31].

Since redox status in G6PD deficiency may be disturbed, there is a potential application of antioxidant dietary supplementation in physically active individuals with this deficiency. To the best of our knowledge, no study has looked into the effects of supplementation on health aspects in G6PD-deficient individuals after exercise. One study by Tsakiris and colleagues [32] investigated the effect of exercise training and alpha-tocopherol supplementation on G6PD activity in erythrocytes of non-G6PD-deficient individuals. Ten basketball players participated in a game (forced training) before and 30 days after alpha-tocopherol supplementation, with blood samples obtained before and immediately after each game. Alpha-tocopherol supplementation resulted in increased pre- and postgame total antioxidant status (TAS). Postgame G6PD activity was also increased after alpha-tocopherol supplementation compared to postgame G6PD activity at the baseline. These results indicate that alpha-tocopherol supplementation could potentially lead to increased antioxidant capacity in G6PD-deficient individuals after a period of strenuous training. Further research is needed to assess whether supplementation with antioxidants or cysteine donors would increase GSH levels and therefore help cells counterbalance exercise-induced oxidative stress, an effect that could benefit G6PD-deficient individuals that participate in exercise.

## 5. Conclusions

In conclusion, the limited available experimental data indicate that G6PD-deficient individuals may safely participate in exercise of various intensities. This conclusion stems from results from full-scale controlled research trials that employed the most rigorous and demanding types of exercise, using a large number of parameters that showed no harmful effects after several modes and intensities of exercise on G6PD-deficient individuals. This is of great importance if someone takes into account that exercise has pleiotropic positive effects on health, and G6PD deficiency is the most common enzymopathy. Excluding individuals with this enzymopathy from participating in exercise a priori or based on some case studies does not seem a prudent thing to do. There are some reports of clinical signs of haemolysis, muscle degeneration, myalgia, and myoglobinuria after heavy exercise in G6PD-deficient individuals that come from case studies, and they should be viewed with caution when interpreting them. Deficient G6PD activity in skeletal muscles and other tissues than RBC observed in some individuals may play a role in this phenomenon. Therefore, future studies should examine the responses to exercise of individuals with different levels of G6PD activity in various tissues. Finally, since GSH levels are lower in G6PD deficiency, it would be interesting to examine the effects of antioxidant or cysteine donor supplements on redox status after acute exercise or chronic training in these individuals.

## Figures and Tables

**Table 1:** Case report studies that examined the effects of exercise on various haematological and/or blood redox status indices in G6PD-deficient individuals.

Study	Subject(s)	Exercise	Symptoms	Results	Comments
Bresolin et al. [15]	30 yrs man, pentathlon-trained athlete	12 km competitive run	Loss of consciousness and pigmenturia during the last meters of run Sweating, subcyanosis, myosis	↓BP, ↑HR, hypoglycaemia, ↑BT, ↑WBCs, metabolic acidosis, jaundice with ↑total & direct bilirubin, ↑SGOT & SGPT, ↑CK, ↑LDH; urine: Hb, myoglobin, ketone bodies; ECG: sinusoidal tachycardia Next day: ↑haemolysis, ↓haptoglobin, ↓Hb, ↓RBC, ↑↑total & direct bilirubin A few months later: Normal bilirubin, SGOT, SGPT, CK, LDH (decreased gradually)	G6PD activity: RBC 0.9%, PTLs 35%, WBCs 16.2%, muscle 1.3%, myoblasts 17.8%, myotubes 18.8%, and skin fibroblasts 18.4% of controls

Kimmick & Owen [16]	34 yrs black man (G6PD deficiency and sickle cell trait; normal muscle G6PD levels)	Vigorous exercise	Severe oxidative haemolysis and rhabdomyolysis 24 hours after vigorous exercise; a total of three similar episodes within 21 months	Malaise, myalgia, myoglobinuria, ↓haptoglobin, bite cells indicating oxidative haemolysis, anaemia	The combination of two red blood cell defects (G6PD deficiency and sickle cell trait) may render RBC more susceptible to oxidative stress, resulting in severe haemolysis after intense exercise

Demir et al. [17]	37 yrs man, elite long distance runner	—	No clinical sign of haemolysis	↓haptoglobin, ↑(slightly) unconjugated bilirubin over the years ↓G6PD activity in RBC, WBCs, muscle	G6PD activity in RBC: ~9% of normal; G6PD activity in leukocytes: ~63% of normal Calculated^∗^ muscle G6PD activity: ~13.7% of normal

BP: blood pressure, HR: heart rate, BT: body temperature, WBCs: white blood cells, Hb: haemoglobin, RBC: red blood cells, SGOT: serum glutamic oxaloacetic transaminase, SGPT: serum glutamic pyruvic transaminase, CK: creatine kinase, LDH: lactate dehydrogenase, ECG: electrocardiogram, PTLs: platelets, EEG: electroencephalogram, Hct: haematocrit, ^∗^equation: *y* = 0.39*x* + 0.198, according to Ninfali et al. [13].

**Table 2:** Experimental studies that examined the effects of exercise on various haematological and/or blood redox status indices in G6PD-deficient individuals.

Study	Subject(s)	Exercise	Symptoms	Results	Comments
Nikolaidis et al. [18]	9 males (29.9 ± 6.1 yrs) with G6PD deficiency (D) & 9 males (31.0 ± 4.0 yrs) with normal G6PD activity (N)	Two exhaustive treadmill exercise protocols of different duration (12 min & 50 min)	No	Preexercise (12 min): ↓RBC, ↓Hct, ↓Hb, ↓GSH, ↓GSSG in D than N Postexercise (12 min): ↑TBARS, ↑PC, ↑catalase, ↑TAC, ↓GSH (both in D and N), ↓RBC, ↓Hct, ↓Hb in D than N Preexercise (50 min): ↓Hct, ↓Hb, ↓GSH, ↓GSSG in D than N Postexercise (50 min): ↑TBARS, ↑PC, ↑catalase, ↑TAC, ↓GSH, ↑GSSG, ↓GSH/GSSG (both in D and N), ↓RBC, ↓Hct, ↓Hb in D than N	Exercise until exhaustion did not lead to higher oxidative stress in D in comparison to N

Jamurtas et al. [19]	9 males (29.1 ± 3.1 yrs) with G6PD deficiency (D) & 9 males (29.0 ± 2.0 yrs) with normal G6PD activity (N)	Run at ~75% HRmax for 45 min	No	Preexercise: ↓Hct, ↓GSH in D than N Postexercise: no changes in GSH, GSSG, GSH/GSSG or lipid hyperoxides (in both D and N), ↓GSH in D than N	45 min of moderate intensity exercise did not increase markers of oxidative stress and haemoglobin oxidation in D or N

Theodorou et al. [20]	9 males (23.3 ± 0.8 yrs) with G6PD deficiency (D) & 9 males (22.6 ± 0.4) with normal G6PD activity (N)	Eccentric muscle-damaging exercise	No	Preexercise: ↓GSH, ↓GSSG in D than N Postexercise (5 days): changes in indices of muscle function, redox status, and haemolysis in D were similar to N; ↓GSH, ↓GSSH in D than N	High-intensity muscle-damaging exercise did not lead to different perturbations of muscle function, blood redox status, and haemolysis in D compared to N

Chanda et al. [21]	18 females (20.7 ± 0.2 yrs) with G6PD deficiency (D), 18 females (21.1 ± 0.2 yrs) with normal G6PD activity (N)	Two treadmill exercise protocols of different intensity (maximal (ME) and 75% HRmax (MM))	No	ME: ↑total MP for 45 minutes after the exercise, increase was higher in D as compared to N MM: no change in total MP Total MP concentrations were inversely correlated with G6PD activity Total MP concentrations were positively correlated with MDA concentrations	D may participate in MM without higher MP concentration and oxidative stress compared to N

Lee et al. [28]	27 yrs man with G6PD deficiency (D), four males with normal G6PD levels (N)	Run at 70-75% HRmax for 30 min	No	Postexercise: ↓GSH in N	No differences between D and N probably due to high aerobic fitness status of D

HR: heart rate, Hb: haemoglobin, RBC: red blood cells, Hct: haematocrit, GSH: reduced glutathione, GSSG: oxidized glutathione, TBARS: thiobarbituric acid reactive substances, PC: protein carbonyls, TAC: total antioxidant capacity, ME: maximal exercise, MM: moderate-intensity exercise, MP: microparticles.
